# Erratum for Shaping Alcohol Health Literacy: A Systematic Concept Analysis and Review

**DOI:** 10.3928/24748307-20200116-01

**Published:** 2020-02-11

The article “Shaping Alcohol Health Literacy: A Systematic Concept Analysis and Review” by Orkan Okan, Gill Rowlands, MBBS, MD, FRCP, FRCGP, Susie Sykes, PhD, and Jane Wills, which was published in the January 2020 issue of *HLRP: Health Literacy Research and Practice* (volume 4, number 1, pp. e3–e20), has been amended to include factual corrections. An error was identified subsequent to its original publication. Figure 4 was not shown in its entirety (the words “subjective knowledge” could not be seen). The online article and its erratum are considered the version of record.

## Figures and Tables

**Figure 4.**
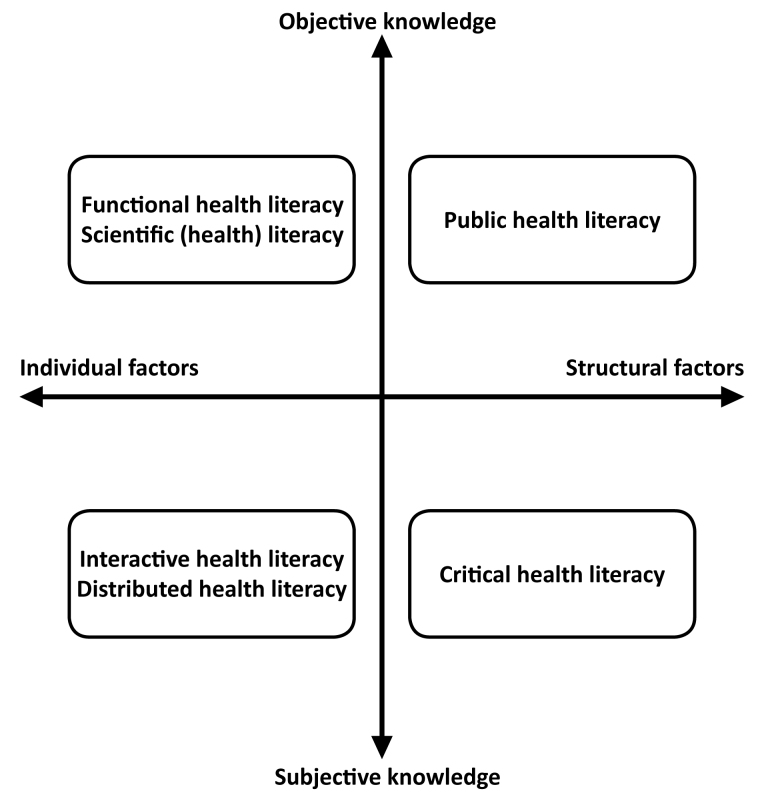
The domains of alcohol health literacy.

